# Varus–valgus stability at 90° flexion correlates with the stability at midflexion range more widely than that at 0° extension in posterior-stabilized total knee arthroplasty

**DOI:** 10.1007/s00402-017-2779-7

**Published:** 2017-08-28

**Authors:** Kazunori Hino, Tatsuhiko Kutsuna, Kunihiko Watamori, Hiroshi Kiyomatsu, Yasumitsu Ishimaru, Jun Takeba, Seiji Watanabe, Yoshitaka Shiraishi, Hiromasa Miura

**Affiliations:** 1 Department of Orthopaedic Surgery, Ehime University Graduate School of Medicine, Shitsukawa, Toon, Ehime 790-0295 Japan; 2 Department of Biomechanics, Translational Research Center, Ehime University Hospital, Shitsukawa, Toon, Ehime 790-0295 Japan

**Keywords:** Total knee arthroplasty, Midflexion, Laxity, Stability, Balance, Soft tissue

## Abstract

**Introduction:**

Midflexion stability can potentially improve the outcome of total knee arthroplasty (TKA). The purpose of this study was to evaluate the correlation between varus–valgus stability at 0° of extension and 90° of flexion and that at the midflexion range in posterior-stabilized (PS)-TKA.

**Materials and methods:**

Forty-three knees that underwent PS-TKA were evaluated. Manual mild passive varus–valgus stress was applied to the knees, and the postoperative maximum varus–valgus stability was measured every 10° throughout range of motion, using a navigation system. Correlations between the stability at 0°, 90° of flexion, and that at each midflexion angle were evaluated using Spearman’s correlation coefficients.

**Results:**

The stability of 0° modestly correlated with that of 10°–20°, but it did not significantly correlate with that of 30°–80°. However, the stability of 90° strongly correlated with that of 60°–80°, modestly correlated with that of 40°–50°, weakly correlated with that of 20°–30°, and did not correlate with that of 10°.

**Conclusions:**

The present study confirmed the importance of acquiring stability at 90° flexion to achieve midflexion stability in PS-TKA. However, initial flexion stability did not strongly correlate with the stability at either 0° or 90°. Our findings can provide useful information for understanding varus–valgus stability throughout the range of motion in PS-TKA. Attention to soft tissue balancing is necessary to stabilize a knee at the initial flexion range in PS-TKA.

## Introduction

Analyses of failure mechanisms following total knee arthroplasty (TKA) show that instability is a commonly identified cause for revision [1–3]. Advantages of adequate soft tissue balancing include enhanced clinical outcomes and postoperative patient satisfaction [3, 4]. In contrast, increased implant wear, pain, loosening, and instability are linked to inadequate soft tissue balance [3, 5–8]. Recently, there has been increased interest in the varus–valgus stability in the coronal plane [9–11]. In addition, activities of daily living cause mechanical load on the knee joint, both in full extension and in midflexion [12]. However, studies on midflexion stability have been limited due to the difficulty of correctly assessing the knee flexion angle, despite its importance. Insufficient assessment of the midflexion stability may have resulted in apparently contradictory conclusions about the association between stability and outcomes in previous studies. Practically speaking, unexpected wear and complications with the post-cam mechanism have been reported [13, 14]. Midflexion stability may improve outcomes and patient satisfaction.

The acquisition of appropriate soft tissue balance is largely dependent on the surgeon’s intraoperative judgment. To avoid instability caused by inappropriate soft tissue balance, a widely recognized goal is to adjust the soft tissue envelope to feel stable in 0° extension and 90° flexion by using a manual stress test, gap spacer, or tension meter. This technique can reliably achieve a more stabilized knee in 0° extension and 90° flexion. However, it is unclear whether it can achieve stability at the midflexion range.

It is hypothesized that intraoperative varus–valgus stability at 0° and 90° has an impact on postoperative varus–valgus stability at the midflexion range, and this impact differs between each degree of flexion. The purpose of this study was to evaluate the correlation between varus–valgus stability at 0° extension and 90° flexion and that at the midflexion range.

## Materials and methods

Forty-three patients underwent TKA with the PS prosthesis (LPS-flex; Zimmer, Warsaw, IN, USA) using a navigation system (precisioN Knee Navigation Software, version 4.0; Stryker, Kalamazoo, MI, USA). To minimize the influences of clinical variables, patients with valgus knee or preoperative severe contracture (>20° or <90°) were excluded. The patient population included 28 women and 15 men with a mean age of 77 ± 4.8 years. The study population was normally distributed. The average preoperative hip knee ankle angle was 14.2° ± 5.5° in varus knees and the postoperative hip knee ankle angle was 0.0° ± 2.0°.

The institutional review board of our university approved this study, and informed consent was obtained from all patients.

### Surgical procedure and varus–valgus stability measurements

The air tourniquet was inflated to 250 mmHg under general anesthesia in all cases intraoperatively, and specific anatomic reference points were located by anchoring infrared signal transducers into the femur and tibia with pins. Then, the skin incision was made, and the subcutaneous tissue was exposed. Registration was performed with osteophytes and soft tissues, including the intact preservation of the anterior cruciate ligament (ACL). The anteroposterior and rotational axes of the femur and tibia were identified based on anatomical landmarks. The femoral rotation axis was defined by referring to the average rotation axis of the axis perpendicular to the white side line and parallel to the transepicondylar axis. The tibial rotation axis was directed along the line from one-third of the tibial tubercle to the central point of the transverse diameter, excluding the osteophytes calculated during preoperative planning.

In the next step, the distal femur was cut using the navigation-assisted measured resection technique; the proximal tibial cut was made using extramedullary alignment rods. After removing the osteophytes, trial components were placed into the knee, and the knee was manually maneuvered throughout flexion to assess soft tissue balance. When the soft tissue balance was inappropriate, the medial collateral ligament, posterior knee capsule, or other tissue was carefully avulsed in an incremental manner for adjustment. After confirming that the TKA components and inserts were firmly placed in an appropriate position, the surgical incision was completely closed. The investigator gently applied physiologically allowable maximal manual varus–valgus stress to the knee without angular acceleration, and the varus–valgus angle of the femorotibial axis was measured automatically by the navigation system every 10° throughout flexion. The results obtained by a single investigator. The navigation system determined the varus–valgus orientation using Euler angles and spatial coordinates, which allowed for the assessment of varus–valgus angulation independent of knee flexion and rotation (positive values indicate varus orientation, and negative values indicate valgus orientation).

### Power sample analysis and validation

To achieve a correlation of *ρ* = 0.5 with 80% power and an *α* = 0.05, it was calculated that a minimum sample size of 30 participants would be required. The accuracy of the navigation system was established at 0.5°. Preliminary investigations confirmed that the test–retest reliability of varus–valgus stress angles indicated that the interclass and intraclass correlation coefficients (ICCs) were sufficiently high, with values >0.8 at each measured knee flexion angle.

### Statistical analysis

Non-parametric tests were used because of the small sample size, although an arithmetically sufficient normal distribution was found. Correlations were evaluated using Spearman’s coefficients of correlation. Analyses were performed with JMP, version 11.0 (SAS Institute, Tokyo, Japan). A *ρ* value >0.7 was considered a strong correlation; 0.4 < *ρ* < 0.7 was considered a modest correlation; and 0.2 < *ρ* < 0.4 was considered a weak correlation. A *P* value < 0.05 was considered statistically significant. Statistical analyses for ICCs were performed using IBM SPSS, version 23 (IBM Corp., Armonk, NY, USA). An ICC >0.8 was considered an almost perfect correlation.

## Results

**Fig. 1:**
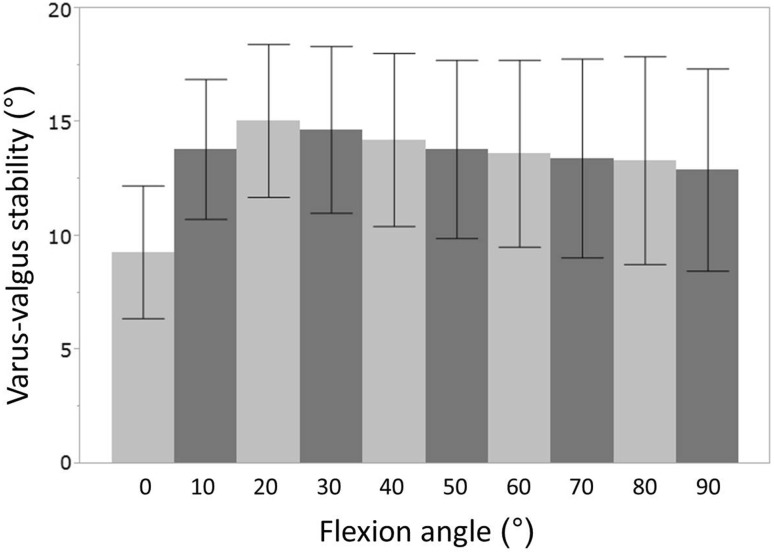
*Bar chart* showing the mean postoperative varus–valgus stability throughout the range of flexion in posterior-stabilized total knee arthroplasty. For both charts *Y axis* varus–valgus stability (°), *X axis* flexion angle (°). *Upper whiskers* indicate the standard deviation

The measurements of postoperative varus–valgus stability throughout range of motion are presented in Fig. 1. Varus–valgus stability increased sharply for the flexion range of 0°–20°, and it changed gradually thereafter.

**Fig. 2:**
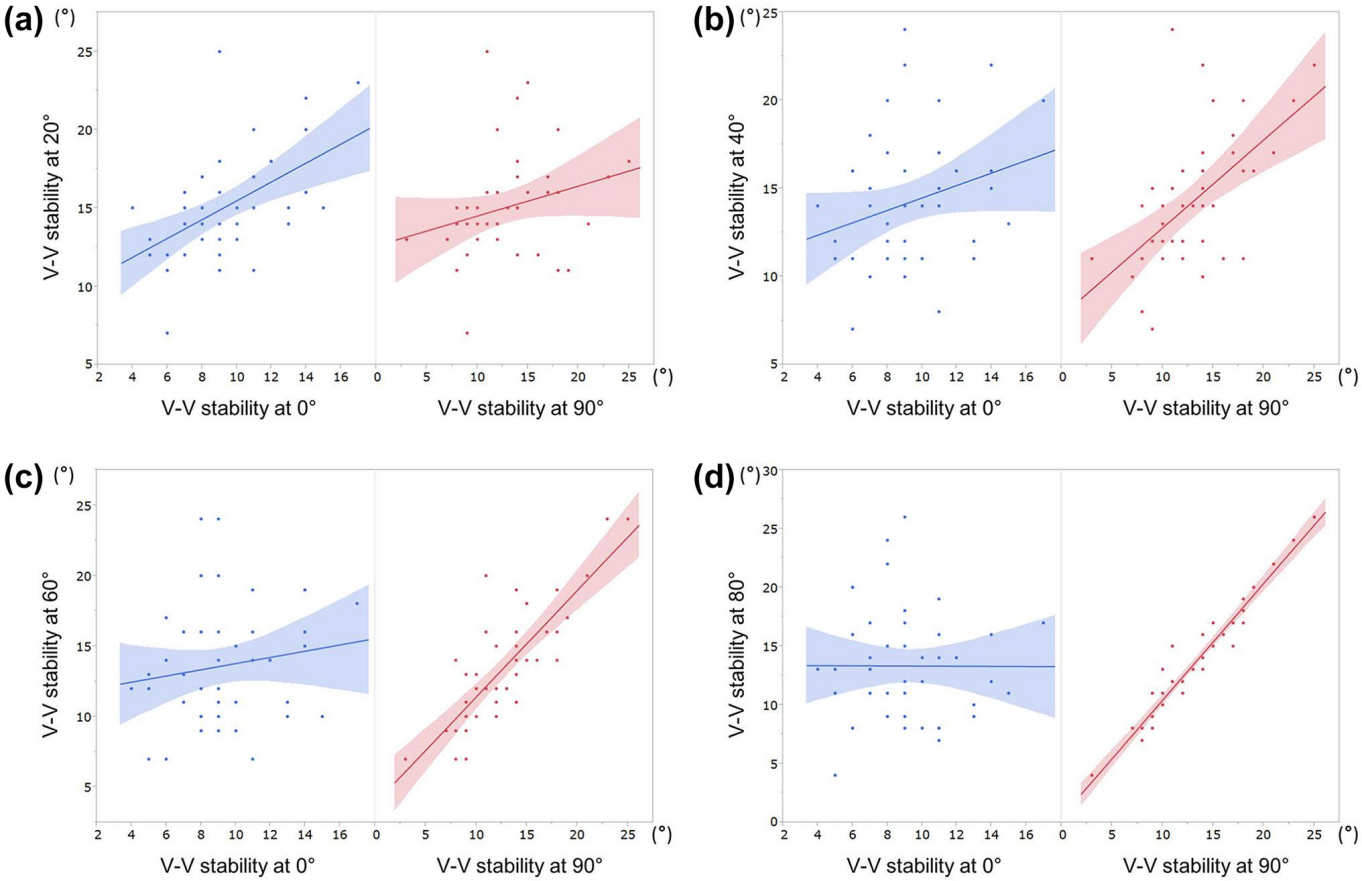
Scatterplots of varus–valgus stability at 0° and 90° (*horizontal axis*) and varus–valgus stability at each midflexion angle (*vertical axis*). **a** 20°, **b** 40°, **c** 60°, **d** 80°

Table 1 shows Spearman’s correlations (*ρ*) between 0°, 90°, and each measured midflexion varus–valgus stability. Results of the assessment of the correlation showed that the stability of 0° modestly correlated with that of 10°–20°, but it did not significantly correlate with that of 30°–80° (Table 1; Fig. 2). However, the stability of 90° strongly correlated with that of 60°–80°, modestly correlated with that of 40°–50°, weakly correlated with that of 20°–30°, and did not correlate with that of 10° (Table 1; Fig. 2).

**Table 1:** Spearman’s correlations (*ρ*) between the varus–valgus stability at 0°, 90°, and that at each midflexion varus–valgus stability

V–V stability	V–V stability	Spearman (*ρ*)	*P* value
0°	10°	0.68	<0.0001*
	20°	0.49	0.001*
	30°	0.29	0.063
	40°	0.22	0.148
	50°	0.24	0.116
	60°	0.14	0.360
	70°	0.11	0.501
	80°	−0.03	0.848
90°	10°	0.03	0.826
	20°	0.34	0.026*
	30°	0.39	0.010*
	40°	0.58	<0.0001*
	50°	0.69	<0.0001*
	60°	0.78	<0.0001*
	70°	0.88	<0.0001*
	80°	0.95	<0.0001*

* *P* < 0.05

Values dividing the amount of varus–valgus stability at 90° by the amount of varus–valgus stability at 0° were significantly correlated with the amount of varus–valgus stability at 10° and 50°–80° (Table 2). However, no significant correlation was observed between the amount of varus–valgus stability at 20°–40° and the values dividing the amount of varus–valgus stability at 90° by the amount of varus–valgus stability at 0° (Table 2).

**Table 2:** Spearman’s correlations (*ρ*) between the value dividing the amount of varus–valgus stability at 90° by that at 0° and the amount of varus–valgus stability at each midflexion range

Value	V–V stability	Spearman (*ρ*)	*P* value
90°/0°	10°	−0.41	0.007*
	20°	−0.07	0.673
	30°	0.08	0.624
	40°	0.26	0.095
	50°	0.33	0.032*
	60°	0.45	0.002*
	70°	0.54	0.000*
	80°	0.67	<0.0001*

* *P* < 0.05

## Discussion

The most important finding of this study was that stability at 0° modestly correlated with stability in the narrow range up to 20°. However, stability at 90° correlated with stabilities in the comparatively wide range from 20° to 80°. This suggests the importance of acquiring stability at 90° flexion to achieve midflexion stability in PS-TKA.

Accurate intraoperative soft tissue evaluation is made possible by recent technological advances, such as the tension meter and navigation system [10, 15–18]. However, clear indicators of soft tissue balance remain elusive. Traditionally, the aim of TKA is to achieve equal medial and lateral gaps as well as equal flexion and extension gaps. Yet, the feasibility of this concept for all cases remains controversial. One reason is that normal knees do not always have equal medial and lateral gaps or equal flexion and extension gaps [19, 20]. Nowakowski et al. demonstrated that extension and flexion gaps are asymmetric and unequal. They also showed that ACL and posterior cruciate ligament (PCL) resections produce varying gap changes using a prototypical force determining ligament balancer without the need for bony resection. The sequential cruciate ligament resections tended to reflect varying TKA designs [21]. The cruciate ligaments, functioning as secondary stabilizers against varus–valgus torque, bear approximately one-fourth of the varus–valgus load of the collateral ligaments [22, 23]. Therefore, in PS-TKA, instability in the midflexion range is suspected to occur. Medial release is a classic method for adjusting soft tissue balance. Complete release of the medial collateral ligament (MCL) increases medial stability to 6.9° in full extension and 13.4° at 90° flexion [24]. Releasing the MCL enlarges the medial flexion gap more than the extension gaps [25]. These results illustrate the difficulty in managing extension imbalance using only medial release.

To avoid instability caused by inappropriate soft tissue balance, a widely recognized goal in PS-TKA is to create symmetric, rectangular gaps (equal medial and lateral gaps as well as equal flexion and extension gaps); this has been named the gap technique. This concept can reliably achieve a more stable knee in 0° extension and 90° flexion. However, it is unclear if this technique can achieve stability at the midflexion range. Minoda et al. reported that the center size of the joint gap was loose, especially at 30° flexion in PS-TKA, even when using the gap technique. The authors postulate that this is probably because in PS-TKA, both the ACL and PCL are sacrificed and a cam-post mechanism regulates the anterior–posterior translation. There is no stabilizer of the anterior–posterior translation in the midflexion range in which the cam-post mechanism does not engage. Incavo et al. [11] examined the relationship between the difference of alignment axes and the implication of medial–lateral soft tissue balance in TKA. Their result indicated that measurements of medial and lateral joint spacing are statistically significantly different at all flexion angles between the two alignments. The anatomic alignment axes’ pattern demonstrates midflexion lateral opening and late-flexion medial joint space opening. On the other hand, mechanical axes have a consistent 2–3 mm larger lateral space than a medial joint space. In addition to these factors, the insert thickness, changes of posterior condylar offset, and design of the prosthesis has been also reported affect midflexion stability [26–28].

The present study showed that varus–valgus stability in the initial flexion range did not strongly correlate with stability at 0° or 90°. We expected a similar correlation at the wide range in midflexion and 0° in extension positions. However, modest correlation was found with 10° and 20°. Moreover, the present study showed that the values dividing the amount of varus–valgus stability at 90° by the amount of varus–valgus stability at 0° were significantly correlated only with the amount of varus–valgus stability at 10° and 50°–80°. No significant correlation was observed with the amount of varus–valgus stability at 20°–40°. These findings indicate that it is not possible to reliably acquire stability at the initial flexion range, even when achieving a stable knee at 0° extension and 90° flexion, and even when achieving equal flexion and extension stability. This suggests the possibility that another element is involved in stabilities at the initial flexion range. In some cases, removing the ACL/PCL in PS-TKA would lead to large stability at the initial flexion range, despite the recovery of proper soft tissue balance in 0° knee extension and 90° knee flexion. Careful attention would be necessary to prevent varus–valgus instability in the initial flexion range in PS-TKA. To adjust the stability of the initial flexion range in PS-TKA, it has been suggested that precise surgical measures, further improvement in the implant design, joint line control, matching implant sizes, and other factors are needed.

One limitation of this study is that it reported on the LPS-flex model only. The measured resection technique was used for bone cutting, and surgery was performed using tourniquet vascularization and non-load-bearing conditions under general anesthesia. Additionally, only patients with varus-type arthritis were treated, and the procedure involved manual passive stress by the operator. Furthermore, stress force was not standardized. These limitations restrict the generalization of our results. Ghosh et al. [10] confirmed that computer navigation can be used to analyze soft tissue balance during TKA beyond the coronal plane and throughout range of motion, and stress test results showed reproducible patterns of soft tissue balance. The strengths of the current study include the use of a navigation system that enabled precise evaluation of varus–valgus position throughout the range of motion. Additionally, evaluating the postoperative outcome at a final state of healing with a strong suture to the capsule and skin was clinically meaningful. Future research is required to determine the degree of varus–valgus stability adequate for TKA procedures. Moreover, further study is required to improve the surgical theory and implant design to reliably achieve full range stability, and to investigate the relationship between midflexion stability and patients’ clinical outcome.

## Conclusions

Varus–valgus stability at 0° extension correlates with narrow, initial flexion range stability. Varus–valgus stability at 90° flexion correlates with stability at a wide midflexion range, confirming its importance in acquiring stability at 90° flexion to achieve midflexion stability. The present results will help orthopedists understand the midflexion stability after PS-TKA.
